# Supplementary material for: Recent Vaccination Against SARS-CoV-2 Is Associated with Less Severe Disease in Working-Age Adults
Source: Int J Environ Res Public Health. 2024 Nov 12;21(11):1501. doi: 10.3390/ijerph21111501 (PMC11594206; doi:10.3390/ijerph21111501)
Supplement: Supplementary file 1 [file ijerph-21-01501-s001.zip › ijerph-3252529-supplementary.pdf]

Supplemental tables for "Recent vaccination against SARS-CoV-2 is associated with less severe disease in working-age adults"

Table S1 Association between recent COVID-19 vaccination and severity of illness *without adjustment for previous infection*; Canadian healthcare and education workers, June 2020-December 2023. Relative risk ratio[1] (95% confidence intervals) estimated by multinomial logistic regression with robust variance estimator

**Focal variable**	**Regular activities (N=1229)**	**Unwell (N=1926)**	**Bed rest (N=602)**
COVID-19 vaccine			
>12 months prior	Reference group	Referent	Referent
None/one of two-dose		0.81 (0.40, 1.61)	0.93 (0.33, 2.60)
6 to 12 months prior		0.86 (0.65, 1.13)	0.86 (0.60, 1.22)
<6 months prior		0.73 (0.54, 0.97)*	0.71 (0.49, 1.03)
**Confounding variables**			
Age in years	Reference group	1.01 (1.00, 1.02)*	1.01 (1.00, 1.02)
Gender: Female	Reference group	Referent	Referent
Male		0.65 (0.52, 0.81)**	0.40 (0.28, 0.57)†
Health status: Poor/fair/good	Reference group	Referent	Referent
Very good		0.76 (0.63, 0.91)*	0.67 (0.53, 0.86)†
Excellent		0.72 (0.59, 0.89)*	0.49 (0.37, 0.66)†
Heart disease: No	Reference group	Referent	Referent
Yes		1.22 (0.67, 2.22)	2.18 (1.06, 4.48)*
SARS-CoV-2 variant period			
Wuhan (L)	Reference group	Referent	Referent
Alpha		1.49 (0.72, 3.11)	1.18 (0.48, 2.91)
Delta		0.65 (0.21, 2.03)	0.20 (0.02, 2.07)
Omicron BA.1		1.43 (0.65, 3.16)	0.65 (0.21, 1.99)
Omicron BA.2		2.14 (0.96, 4.76)	1.60 (0.52, 4.94)
Omicron BA.4/5		1.73 (0.77, 3.90)	1.16 (0.37, 3.59)
Recombinant		1.93 (0.84, 4.41)	1.28 (0.40, 4.06)

* p<0.05; † p≤0.001

[1)] Adjusted for study (healthcare vs education worker), >1 illness from the same participant, and other variables in table

Table S2 Association between recent COVID-19 vaccination and severity of illness *symptomatic infections only* (N=3634); Canadian healthcare and education workers, June 2020-December 2023. Relative risk ratio[1] (95% confidence intervals)

**Focal variable**	**Regular activities (N=1106)**	**Unwell (N=1926)**	**Bed rest (N=602)**
COVID-19 vaccine[2]			
>12 months prior	Reference group	Referent	Referent
None/one of two-dose		0.83 (0.39, 1.76)	0.94 (0.33, 2.72)
6 to 12 months prior		0.82 (0.62, 1.09)	0.81 (0.56, 1.16)
<6 months prior		0.69 (0.51, 0.93)*	0.67 (0.45, 0.98)*
**Confounding variables**			
Previous COVID-19 infection[2]			
>12 months prior	Reference group	Referent	Referent
None		1.28 (0.97, 1.68)	1.35 (0.93, 1.95)
6 to 12 months prior		0.95 (0.65, 1.38)	0.65 (0.38, 1.12)
<6 months prior		0.59 (0.35, 0.99)*	0.77 (0.38, 1.56)
Age in years	Reference group	1.01 (1.00, 1.02)*	1.01 (1.00, 1.02)*
Gender: Female	Reference group	Referent	Referent
Male		0.65 (0.52, 0.82)†	0.41 (0.28, 0.59)†
Health status: Poor/fair/good	Reference group	Referent	Referent
Very good		0.77 (0.63, 0.93)*	0.68 (0.53, 0.87)*
Excellent		0.73 (0.58, 0.90)*	0.49 (0.37, 0.67)†
Heart disease: No	Reference group	Referent	Referent
Yes		1.83 (0.91, 3.69)	3.21 (1.47, 7.01)*
SARS-CoV-2 variant period			
Wuhan (L)	Reference group	Referent	Referent
Alpha		1.26 (0.55, 2.87)	1.00 (0.38, 2.66)
Delta		0.55 (0.16, 1.87)	0.18 (0.02, 1.97)
Omicron BA.1		1.00 (0.41, 2.44)	0.45 (0.14, 1.47)
Omicron BA.2		1.46 (0.60, 3.59)	1.09 (0.34, 3.57)
Omicron BA.4/5		1.20 (0.48, 2.97)	0.82 (0.25, 2.69)
Recombinant		1.40 (0.56, 3.54)	0.98 (0.29, 3.33)

* p<0.05; † p≤0.001

1) Adjusted for study (healthcare vs education worker), >1 illness from the same participant, and other variables in table

2) ≥10 days prior to date of [current] infection

Table S3 Association between number of solicited symptoms reported and recency of COVID-19 vaccination; Canadian healthcare and education workers, June 2020-December 2023. Coefficient (95% confidence intervals)

**Focal variable**	**Estimate[1]**
COVID-19 vaccine[2]	
>12 months prior	Referent
None/one of two-dose	-0.52 (-1.73, 0.70)
6 to 12 months prior	-0.22 (-0.63, 0.19)
<6 months prior	-0.58 (-1.02, -0.14)*
**Confounding variables**	
Previous COVID-19 infection[2]	
>12 months prior	Referent
None	0.39 (-0.03, 0.81)
6 to 12 months prior	-0.27 (-0.81, 0.27)
<6 months prior	-0.54 (-1.33, 0.26)
Age in years	-0.02 (-0.03, -0.01)*
Gender: Female	Referent
Male	-0.85 (-1.20, -0.50)†
Health status: Poor/fair/good	Referent
Very good	-0.80 (-1.09, -0.52)†
Excellent	-1.20 (-1.52, -0.88)†
Heart disease: No	Referent
Yes	0.68 (-0.33, 1.68)
SARS-CoV-2 variant period:	
Wuhan (L)	Referent
Alpha	0.20 (-1.11, 1.50)
Delta	-0.22 (-2.15, 1.72)
Omicron BA.1	0.34 (-1.10, 1.77)
Omicron BA.2	1.08 (-0.37, 2.52)
Omicron BA.4/5	0.94 (-0.51, 2.39)
Recombinant	1.21 (-0.28, 2.69)

* p<0.05; † p≤0.001

1) Adjusted for study (healthcare vs education worker), >1 illness from the same participant, and other variables in table

2) ≥10 days prior to date of [current] infection

Table S4 Crude associations between illness severity and symptom(s) reported; Canadian healthcare and education workers, June 2020-December 2023. Number (percent)

Symptom	Regular activities (N=1229)	Unwell (N=1926)	Bed rest (N=602)	p-value
Generally unwell	428 (34.8)	1494 (77.6)	509 (84.6)	<0.001
Fatigue	337 (27.4)	1216 (63.1)	477 (79.2)	<0.001
Headache	388 (31.6)	1167 (60.6)	462 (76.7)	<0.001
Coryza	732 (59.6)	1490 (77.4)	459 (76.3)	<0.001
Sore throat/laryngitis	708 (57.6)	1426 (74.0)	452 (75.1)	<0.001
Cough	586 (47.7)	1360 (70.6)	443 (73.6)	<0.001
Myalgia	249 (20.3)	1025 (53.2)	421 (69.9)	<0.001
Fever	103 (8.4)	551 (28.6)	285 (47.3)	<0.001
Anorexia	92 (7.5)	496 (25.8)	277 (46.0)	<0.001
Sinus pain	207 (16.8)	644 (33.4)	274 (45.5)	<0.001
Arthralgia	87 (7.1)	402 (20.9)	213 (35.4)	<0.001
Anosmia and/or ageusia	105 (8.5)	361 (18.7)	186 (30.9)	<0.001
Dyspnea	84 (6.8)	368 (19.1)	181 (30.1)	<0.001
Chest pain	69 (5.6)	305 (15.8)	157 (26.1)	<0.001
Diarrhea	56 (4.6)	279 (14.5)	134 (22.3)	<0.001
Nausea	53 (4.3)	239 (12.4)	132 (21.9)	<0.001
Ear ache	64 (5.3)	265 (13.8)	118 (19.7)	<0.001
Confusion	9 (0.7)	87 (4.5)	73 (12.1)	<0.001
